# Evaluation of the Effect of Management of Drug-Related Problems on Clinical Outcomes of Pulmonary Embolism Outpatients: A Randomized Controlled Trial

**DOI:** 10.3390/jcm14041202

**Published:** 2025-02-12

**Authors:** Yunus Emre Ayhan, Müzeyyen Aksoy, Yahya Abdulrahman, Zeynep Safiye Şahin Eröksüz, Duygu Vezir, Berre Mercümek, Muhammed Yunus Bektay, Sait Karakurt, Mesut Sancar

**Affiliations:** 1 Department of Clinical Pharmacy, Prof. Dr. Cemil Taşcıoğlu City Hospital, 34384 Istanbul, Türkiye; eczyunusemreayhan@gmail.com; 2 Department of Clinical Pharmacy, Faculty of Pharmacy, Marmara University, 34722 Istanbul, Türkiye; muzeyyen.aksoy@marmara.edu.tr (M.A.); sancarmesut@yahoo.com (M.S.); 3 Department of Pulmonary Diseases and Intensive Care, Gaziantep City Hospital, 27470 Gaziantep, Türkiye; yahya.ali.ar@gmail.com; 4 Department of Pulmonary Medicine, Yedikule Teaching and Research Hospital, 34020 Istanbul, Türkiye; zeyneperoksuzmd@gmail.com; 5 Department of Pulmonary and Critical Care Medicine, Sureyyapasa Teaching and Research Hospital, 34844 Istanbul, Türkiye; duyguvezir01@gmail.com; 6 Department of Clinical Pharmacy, Institute of Health Sciences, Bezmialem Vakif University, 34093 Istanbul, Türkiye; 7 Department of Clinical Pharmacy, Faculty of Pharmacy, Bezmialem Vakif University, 34093 Istanbul, Türkiye; 8 Department of Clinical Pharmacy, Faculty of Pharmacy, İstanbul University-Cerrahpaşa, 34500 Istanbul, Türkiye; 9 Department of Pulmonary and Critical Care, School of Medicine, Marmara University, 34722 Istanbul, Türkiye; saitkarakurt@hotmail.com

**Keywords:** pulmonary embolism, bleeding, clinical pharmacist, drug-related problems, anticoagulation

## Abstract

**Background:** Pulmonary embolism (PE) poses significant morbidity and mortality risks, necessitating tailored anticoagulant therapy. Limited studies investigate the drug-related problems (DRPs) in PE. This study aims to evaluate the impact of clinical pharmacist (CP) interventions on drug-related problems (DRPs) and clinical outcomes in outpatients with PE. By addressing DRPs in this specific population, the study seeks to assess the role of CP-led interventions in enhancing patient safety and optimizing treatment outcomes. **Methods:** A randomized controlled trial was conducted on PE patients at a pulmonology outpatient clinic over the period of 15 January 2022 to January 2023. In this trial, the intervention group (IG) benefited from CP recommendations targeting DRPs, while the control group (CG) was observed without any additional interventions. Follow-ups were conducted at 90 and 180 days post-discharge. The study focused on DRPs, CP interventions, and patient outcomes. Data were systematically recorded and analyzed, adhering to ethical standards and employing the PCNE v9.1 classification system. **Results:** The study followed 50 PE patients, with 26 in the IG and 24 in the CG. DRPs, mostly in drug and dose selection, affected 84% of participants (3.34 DRPs/patient). In the IG, the CP made 76 recommendations (91.5%) for 83 DRPs at the prescribing physician level. Majority of these recommendations (94.7%) were accepted. At the 90-day follow-up, bleeding occurred in 16.6% of the CG, with none in the IG (odds ratio [OR] = 2.3, 95% confidence interval [CI]: 1.654–3.198, *p* = 0.046). As indicated by Cramér’s V (0.307), the effect size demonstrated a moderate association between the intervention and the absence of bleeding events in the IG. At the 180-day follow-up, bleeding was 33.3% in the CG and 16.6% in the IG (*p* = 0.443). **Conclusions:** PE patients experience common and frequent DRPs, especially in anticoagulation therapy. CP recommendations are widely accepted but need to be better implemented. No bleeding events were observed in the IG where a CP was involved at 90 days, unlike the CG. Including a CP in the PE treatment team seems to influence outcomes positively.

## 1. Introduction

Pulmonary embolism (PE) is a life-threatening condition caused by thrombotic obstruction of the pulmonary artery, which can lead to significant morbidity and mortality if left untreated [1,2]. Anticoagulant therapy is the cornerstone of PE management and carries a significant risk of severe bleeding, especially when not tailored to individual risk factors [3]. Current guidelines highlight the need to adjust treatment duration according to transient or persistent risk factors to ensure a balance between efficacy and safety [1,2,3]. Anticoagulants such as vitamin K antagonists (VKAs), novel oral anticoagulants (NOACs), and low-molecular-weight heparins (LMWHs) are commonly used in PE treatment. Each drug class has distinct challenges: VKAs have a narrow therapeutic range, LMWHs are associated with adherence issues, and NOACs pose a risk of missed doses due to their short half-lives [1,3,4]. These complexities increase the likelihood of drug-related problems (DRPs), which can adversely affect clinical outcomes.

Despite advances in anticoagulant therapy, research on DRPs in PE patients—especially in outpatient settings—remains limited. Previous studies, such as those analyzing medication errors associated with NOACs or pharmacist interventions in warfarin management, highlight the pivotal role of pharmacists in mitigating adverse events through patient education, dose adjustments, and monitoring [5,6]. However, most of these studies focus on specific anticoagulant classes or general patient populations, leaving a critical gap in understanding the role of clinical pharmacists (CPs) in managing DRPs among PE patients.

This study addresses this gap by evaluating the impact of CP interventions on DRPs and clinical outcomes in PE outpatients. Unlike prior research, which has largely focused on individual anticoagulant classes or broader patient groups, our study specifically tar-gets PE patients in outpatient settings, where DRPs are often underrecognized and under-treated. By focusing on this population, we aim to provide novel insights into the effectiveness of CP-led DRP management in improving patient safety and optimizing care.

The results of this study have the potential to influence clinical practice by demonstrating the value of CP interventions in reducing DRPs and enhancing outcomes for PE patients. Moreover, our findings may inform policy decisions by emphasizing the necessity of structured CP involvement in PE management, particularly in outpatient settings. This study thus contributes to the growing body of evidence supporting the integration of CPs into multidisciplinary care teams for complex conditions like PE.

## 2. Materials and Methods

### 2.1. Setting and Patient Characteristics

This study, designed as a randomized controlled trial, was conducted in the pulmonology department of a tertiary-care university hospital in Istanbul, Türkiye, from 15 January 2022 to 15 January 2023. The inclusion criteria required patients to have a confirmed diagnosis of PE, as specified by the ICD-10 code I26.0. Participants were further required to attend the chest ward outpatient clinic post-discharge and provide informed consent for study participation. Participants were randomly assigned to either the control group (CG) or the intervention group (IG) using the Research Randomizer^®^ software (Version 1.2, Institute for Medical Informatics, Germany), ensuring a 1:1 allocation. A CP prepared a confidential allocation plan by generating stratified sequences linked to consecutive participant numbers. As each participant joined the study, the CP assigned them the next available number, thereby determining their group allocation.

In the IG, the CP’s recommendations concerning DRPs were actively communicated to the healthcare team. Meanwhile, the CG included patients for whom no recommendations were shared, and the care process remained purely observational. Evaluation of patients in both groups occurred at the 90th and 180th days post-discharge, either during outpatient clinic visits or through telephone contact.

Eligible participants were those admitted to the chest diseases service with a verified diagnosis of PE (ICD-10 code: I26.0), who attended post-discharge follow-up at the outpatient clinic and were 18 years or older. The exclusion criteria encompassed patients with a documented history of bleeding, those who experienced death by the 90th day of follow-up, and individuals who were lost to follow-up within this period.

The study was designed to comprehensively evaluate the impact of CP interventions on the clinical outcomes of PE patients.

### 2.2. Sample Size

The sample size for this research was calculated using literature-derived data, showing that CP recommendations resulted in a mean reduction of one DRP per patient [7]. The standard deviation was assumed to be 1. Using these parameters, the effect size (d) was calculated as 0.6062. The significance level (alpha, α) was set at 0.05, and the power of the study (beta, β) was targeted to be 0.80. According to these calculations, the study was designed to achieve a power of 0.8094, with a sample size of 25 patients per group. This sample size was determined to adequately detect the anticipated effect size and achieve statistically significant results in evaluating the impact of CP recommendations on reducing DRPs in patients with PE.

### 2.3. Data Collection

Comprehensive and confidential documentation was ensured throughout the study, covering patient contact information, medical and medication histories, ongoing treatments, laboratory findings, and data essential for evaluating Wells, Geneva, PESI, sPESI, and DASH scores. These details were carefully documented to ensure strict confidentiality and adherence to ethical standards. Data on quality of life for both IG and CG patients—including bleeding incidents, hospitalizations, disease prognosis, treatment outcomes, mortality outcomes, and symptoms such as shortness of breath, pain, and mobility limitations—were systematically collected. Interactions with patients and data collection occurred at the 90th and 180th days post-admission, drawing upon patient charts, the hospital information system, and self-reports.

The research addressed DRPs by analyzing their causes, CP recommendations for their resolution, and the acceptance and application of these recommendations by physicians. For the identification of DRPs, the Turkish adaptation of the Pharmaceutical Care Network Europe Association (PCNE) Classification v9.1 was employed. This validated classification system, developed by the PCNE working group, includes comprehensive domains such as the identification of problems, causal analysis, intervention planning, acceptance levels of proposed interventions, and the resolution status of the issues. Additionally, the classification provides grouped sub-domains, offering an in-depth explanation of its principal domains. The PCNE tool was instrumental in providing a structured approach to DRP identification and management (https://www.pcne.org/upload/files/417_PCNE_classification_V9-1_final.pdf, accessed on 15 January 2022).

This study adhered to the guidelines outlined in the Consolidated Standards of Reporting Trials (CONSORT) [8].

### 2.4. Evaluation of Drug-Related Problems and Clinical Pharmacist Interventions

Patient data for individuals admitted to the chest wards with a confirmed diagnosis of PE were systematically and meticulously documented during their hospital stay. Upon discharge from the chest wards, patients received LMWH treatment and were subsequently recalled for an outpatient clinic follow-up after a two-week interval. During this outpatient clinic follow-up, physicians evaluated the patients’ treatment and planned appropriate anticoagulant therapy. The CP played a crucial role in this process, closely monitoring outpatient follow-ups, implementing necessary treatment modifications, and identifying DRPs through a comprehensive analysis of patients’ medication data.

In this study, we defined a DRP as an event or a circumstance related to drug therapy that actually or potentially interferes with the attainment of desired health outcomes [9]. In response to these challenges, within the IG group, the CP provided in-person recommendations on DRPs to the attending physician, while an experienced CP and a pulmonologist collaboratively assessed the clinical significance of these DRPs. The recommendations did not encompass procedural interventions but were specifically tailored to identifying and preventing DRPs in patients hospitalized for PE and subsequently followed up with in outpatient clinics. These recommendations included initiating or discontinuing specific medications, suggesting therapeutic alternatives, adjusting the route of administration, advocating for therapeutic drug monitoring, optimizing dosage regimens, mitigating potential drug interactions, and refining treatment durations.

Patients in both groups were evaluated during outpatient clinic visits or through telephone follow-ups at the 90th and 180th days post-discharge. Adherence to CP recommendations was monitored through regular outpatient clinic visits, telephone follow-ups, or inquiries made during these evaluations. The implementation status was confirmed by reviewing patient medical records and obtaining direct feedback from physicians regarding the integration of the recommendations into treatment plans.

Potential drug–drug interactions (pDDIs) were evaluated using the Lexicomp^®^ database (Wolters Kluwer Health, Inc., Philadelphia, PA, USA), with only those classified as “major” or “contraindicated” being recognized as DRPs associated with pDDIs. Contraindicated interactions necessitated the avoidance of specific drug combinations, while major interactions prompted consideration of a change in treatment. This meticulous approach aimed to ensure the judicious and safe use of medications in PE treatment.

The recommendations for anticoagulant treatment in patients with PE were derived from authoritative sources, with the primary references being the 2019 ESC Guidelines for the diagnosis and management of acute PE, developed in collaboration with the European Respiratory Society (ERS) [1]. Additionally, guidance was sought from the American Society of Hematology’s 2020 guidelines for managing venous thromboembolism (VTE), explicitly addressing the treatment of deep vein thrombosis. PE served as another crucial source of information [2]. Furthermore, the Turkish Thoracic Society Diagnosis and Consensus Report from 2021 and the Antithrombotic Therapy for VTE Disease Second Update of the CHEST Guideline and Expert Panel Report were selected as additional authoritative references [3,4]. In the current study, these well-established guidelines collectively formed the basis for ensuring the appropriateness and adherence to best practices in anticoagulant therapy for patients diagnosed with PE.

### 2.5. Main Outcome Measure

The study’s primary outcomes centered on identifying and categorizing DRPs, assessing the acceptance rates of CP recommendations, and evaluating complications, including PE recurrence, bleeding, and quality of life indicators, at 90 and 180 days. These outcomes served as key metrics for evaluating the effectiveness of CP interventions in patient management and clinical outcomes. Secondary outcomes involved a comparison of PE patients in terms of disease progression, rates of rehospitalization, and mortality. This secondary analysis sought to provide a holistic understanding of the impact of CP interventions on the disease trajectory and patient outcomes beyond the acute care setting. By comprehensively analyzing both primary and secondary outcomes, the study aimed to contribute meaningful insights into the potential of CP interventions to optimize care, enhance clinical outcomes, and influence the long-term prognosis of PE patients.

Bleeding that was life-threatening or required the transfusion of at least two units of blood was considered major bleeding.

### 2.6. Statistical Analysis

The study used descriptive statistics to present the central tendency and variability of continuous variables, including mean, median, standard deviation, interquartile range (IQR), or count and percentages, as appropriate. For categorical variables, frequency and percentages were provided. Normality testing for continuous variables was performed using the Kolmogorov–Smirnov test. Group comparisons for continuous variables were conducted using either independent t-tests or Mann–Whitney U tests, based on the data’s distributional properties. Associations between categorical variables were assessed with Chi-square tests. For significant Chi-square results, effect size was calculated using Cramér’s V, which quantifies the strength of the association. Effect sizes were interpreted using the following thresholds: small (0.1–0.3), medium (0.3–0.5), and large (>0.5), in accordance with established guidelines [10]. Statistical significance was defined as a *p*-value below 0.05, with a confidence interval (CI) of 95%. Univariate logistic regression analysis was performed to identify significant variables with a threshold of *p* < 0.20, and those significant variables were included in binary logistic regression analysis. Missing data were excluded from the analysis, and the entire dataset was analyzed using IBM SPSS Statistics for Windows, Version 25 (Armonk, NY, USA: IBM Corp.).

Baseline characteristics, including age, sex, and Charlson Comorbidity Index scores, were compared between groups to assess homogeneity and minimize the impact of potential confounding factors. Since no significant differences were found in most parameters, statistical adjustments were not deemed necessary. However, the difference in the number of comorbidities (*p* = 0.028) was taken into account when interpreting the results.

## 3. Results

**Figure 1:**
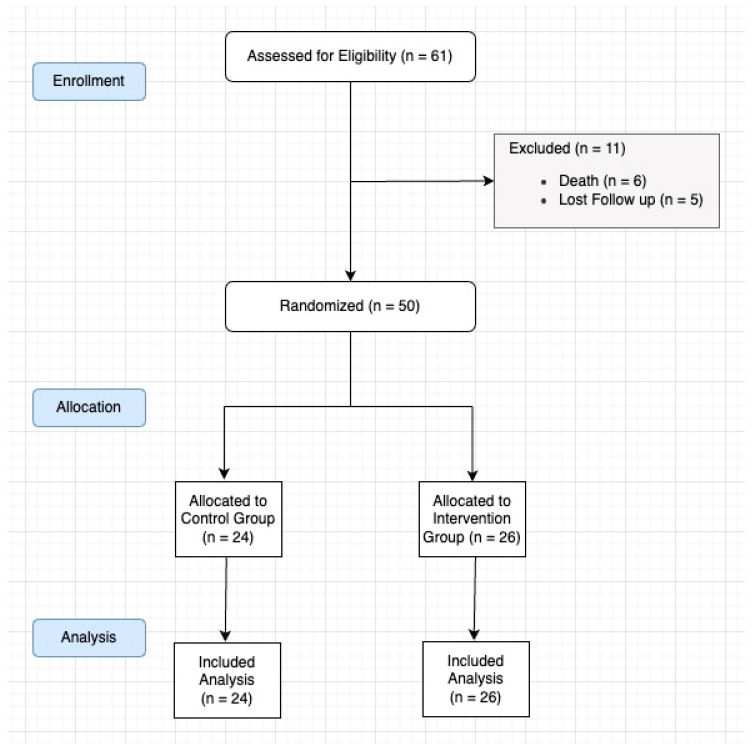
Study flow chart.

During the study period, 61 patients were initially followed up with. Six patients had died, and five were excluded due to loss to follow-up. Consequently, the final cohort comprised 50 patients, with 24 randomly assigned to the CG and 26 to the IG (Figure 1).

Regarding gender distribution, the CG had 16 female patients (66.7%), while the IG had 14 female patients (53.8%). The median age (IQR) of patients in the CG was 69 years (59.25–79), and in the IG, it was 66.5 years (49.75–74.50). Most patients in both groups had at least one comorbidity besides PE. The median (IQR) number of comorbidities per patient was three (2–4) in the CG and two (1–3) in the IG (*p* = 0.028). Hypertension (42%) and diabetes mellitus (26%) were the most prevalent comorbidities in both groups. The median (IQR) DASH scores, indicating the risk of VTE recurrence, were 2 (2–2) in the CG and 3 (2–3) in the IG (*p* = 0.016). Additional sociodemographic and score information is provided in Table 1.

After outpatient follow-up, treatment was consistently administered with LMWHs in most cases (54%). Notably, during the 90-day follow-up on anticoagulation treatment, bleeding incidents were observed in four patients (16.6%) in the CG, while none were reported in the IG (odds ratio [OR] = 2.3, 95% CI: 1.654–3.198, *p* = 0.046). The effect size, as measured by Cramér’s V, was 0.307, indicating a moderate association between the intervention and the reduction in bleeding events. In the CG, bleeding during the 90-day follow-up included hematuria, nosebleeds, petechiae on the arms, and blood in the sputum. These bleeding events were associated with enoxaparin in three patients and edoxaban in one patient. During the 90-day follow-up, no patient developed major bleeding. At the 180-day follow-up, bleeding was observed in 33.3% of patients in the CG and 16.6% in the IG (*p* = 0.443). Throughout the 180-day monitoring period, occurrences of petechiae on the arms (1 instance), blood in the sputum (1 instance), nosebleeds (1 instances), internal bleeding of unspecified location (1 instance), bleeding/bruising at the injection site (2 instances), gastrointestinal bleeding (1 instance), gum bleeding (1 instance), and hemoptysis (1 instance) were noted at the designated frequencies. At the 180-day follow-up, bleeding events were observed in patients receiving warfarin (n = 4), enoxaparin (n = 4), and edoxaban (n = 1). Among these patients, one patient in the CG developed major bleeding. Hospital readmission rates at 90 and 180 days of follow-up were higher in the CG (16.6%, 27.7%, respectively) compared to the IG (15.3%, 11.1%, respectively), although these differences were not statistically significant (*p* > 0.05). On the 180th day, the two groups had no statistically significant difference in mortality rates (*p* = 0.745). Patients in the IG experienced improvements in quality-of-life indicators, with fewer reports of dyspnea, difficulty walking, and pain at 180 days compared to the CG. Although these differences were not statistically significant, they suggest a trend toward enhanced patient outcomes with clinical pharmacist involvement (Table 2).

In the study cohort, at least one DRP was identified in 47 patients, constituting 84% of the patients. The total number of DRPs identified across the CG and the IG was 167, averaging 3.34 DRPs per patient. Specifically, 84 DRPs were identified in the CG, resulting in an average of 3.5 DRPs per patient, while 83 DRPs were identified in the IG, averaging 3.2 DRPs per patient. The most prevalent causes of DRPs were related to drug selection (50.2%) and dose selection (22.1%). Notably, the most common specific issues included the use of medication without indication (C1.2) (22.7%), low dosing (C3.1) (13.1%), and the absence of treatment for an existing indication (C1.5) (10.7%). The distribution of the causes of all DRPs in both groups is detailed in Table 3. Notably, no statistically significant difference was observed between the DRP items and the number of DRPs in both groups (*p* > 0.05).

**Figure 2:**
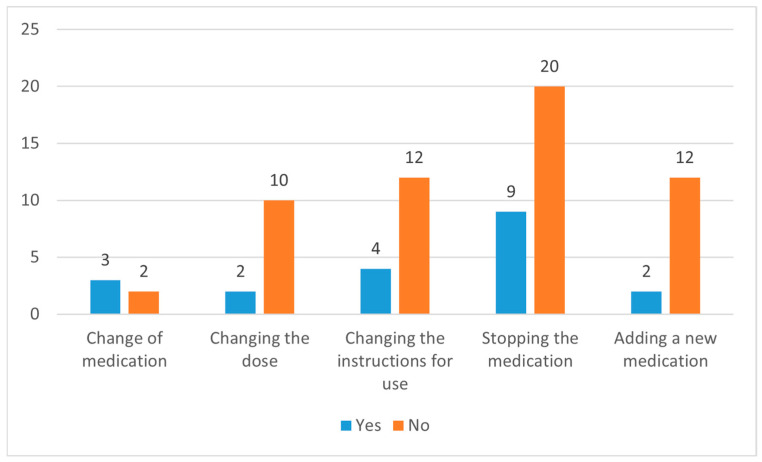
Implementation status of clinical pharmacist recommendations by intervention type.

The CP made 76 recommendations in the IG, addressing 83 DRPs through face-to-face interactions with the prescriber. Among these recommendations, 29 pertained to discontinuing the drug (I3.5) (38.1%), 16 involved changing the instructions for use (I3.4) (21%), 14 suggested adding a new drug (I3.6) (18.4%), 12 proposed altering the dose (I3.2) (15.7%), and 5 recommended replacing the drug with another (I3.1) (6.5%). Remarkably, the prescribers accepted a high percentage of these suggestions—precisely, 72 out of 76 (94.7%). However, implementing the accepted recommendations was notably lower, with only 20 (27.7%) of the accepted recommendations being executed. Among the 52 recommendations that were accepted but not implemented, 24 were related to drug selection, 12 were attributed to dose selection, and 16 were due to other reasons. When the implementation status of the 76 recommendations made by the CP for resolving DRPs was evaluated, it was observed that recommendations for stopping a medication, adding a new medication, and changing the instructions for use were mostly not implemented (Figure 2).

A total of 16 pDDIs, constituting 16.4% of all identified DRPs, were acknowledged as significant in both the CG and the IG. The details and examples of pDDIs is given Table 4. Among these pDDIs, 2 (12.5%) were categorized as contraindicated, while 14 (87.5%) were classified as major-level interactions. The level of evidence for 15 of these pDDIs was deemed “fair,” and one was rated as “excellent.” The most frequently detected major-level interaction pairs were enoxaparin–sertraline (2) and enoxaparin–escitalopram (2), each with their respective frequencies. Additionally, contraindicated-level interaction pairs included escitalopram–rasagiline (1 instance) and pantoprazole–cefuroxime (1 instance). Among all pDDIs, a total of 10 (10/16) pDDIs in eight patients (16%) warranted an “increased antiplatelet effect/increased bleeding risk” warning. However, no significant difference was observed when analyzing the bleeding status of patients with pDDIs carrying a bleeding risk warning compared to those without such pDDIs over 90 days (*p* > 0.05).

**Figure 3:**
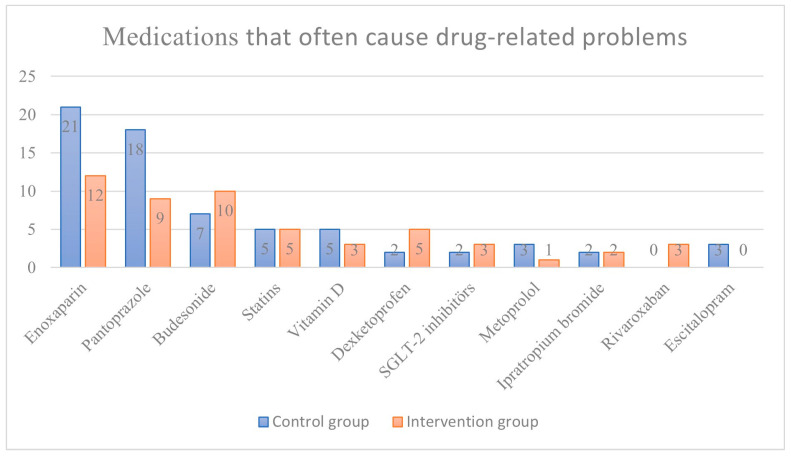
Drugs that cause drug-related problems and their frequency distribution.

The active substances most frequently associated with DRPs were enoxaparin (33/167), pantoprazole (27/167), and budesonide (17/167), combining their frequencies across both groups. Specifically, in the CG, the leading agents causing DRPs were enoxaparin (21/167), pantoprazole (18/167), and budesonide (7/167), while in the IG, enoxaparin (12/167), pantoprazole (10/167), and budesonide (9/167) were identified as the primary causative agents. The distribution of other active substances based on the groups is visually presented in Figure 3.

Upon analyzing the relationship between the 90-day bleeding status of patients and various factors through univariate logistic analysis, it was discerned that the number of comorbidities (*p* = 0.027, R^2^ = 0.25) and the decrease in Pulmonary Embolism Severity Index (PESI) score (*p* = 0.039, R^2^ = 0.25) significantly influenced the 90-day bleeding status. However, no significant model could be established in binary logistic analysis.

## 4. Discussion

In this study, DRPs were systematically compared in terms of disease and drug-related complications, including PE recurrence, bleeding, drug-related adverse events, disease progression, rehospitalization, and mortality rates. These complications were evaluated over 90 and 180 days. This comparative analysis was conducted between the PE patients in the IG, where CP recommendations for DRPs were implemented, and in the CG, where only observation without specific interventions was carried out. Given the limited research on DRPs in PE patients, this study is expected to contribute valuable insights into the role of CPs in mitigating drug-related issues and optimizing patient care outcomes in PE management.

The findings of this study underscore the critical role of CPs in mitigating DRPs and improving patient outcomes. CP involvement not only enhances the optimization of medication regimens but also facilitates interdisciplinary collaboration within hospital settings. This collaborative approach can lead to more coordinated patient care, reduced treatment-related complications, and improved safety measures for patients with complex conditions such as PE. Strengthening the integration of CPs into multidisciplinary teams could further amplify these benefits and establish a model for future practice.

The prevalence of comorbidities, age distribution, and gender representation in patients with PE align closely with those observed in chest wards and internal medicine wards. In congruence with these populations, approximately 60–70% of patients present with three or more comorbidities [7,11,12]. A limited number of studies have explored DRPs in outpatients within chest wards. In a randomized controlled trial conducted by Bektay et al. in chest ward inpatients, 22% of the patients were diagnosed with PE, and at least one DRP was identified in 64.2% of all patients, averaging 1.4 DRPs per patient. The predominant causes of DRPs in both groups were related to drug selection and dose selection. Recommendations for addressing DRPs were accepted in 64.4% of cases [7].

When compared to studies conducted in internal medicine wards, Ayhan and Sancar reported that at least one DRP was identified in 62% of patients (1.59 DRPs per patient). Similar to other studies, DRPs were predominantly attributed to drug selection (76.19%) and dose selection (14.8%). pDDIs (55.78%), errors in dose-timing instructions (9.52%), and inappropriate drug use based on guidelines (8.16%) were the leading causes of DRPs. The most frequently implicated active ingredients were quetiapine (7.72%), tramadol (6%), calcium carbonate (5.57%), methylprednisolone (4.29%), aspirin (3.86%), and pantoprazole (3.43%). The healthcare team accepted 65% of CP resident recommendations for DRPs. However, only 51.25% were fully implemented. Interventions primarily involved discontinuation of the drug (35%) and modification of the instructions for drug use (30%) [12].

In other studies conducted in internal medicine and gastroenterology wards in Türkiye, the number of DRPs per patient has been reported as 1.6–1.7 [7,11]. The prevalence of at least one DRP has been reported to range from 15.4% to 80% across various studies [13,14]. The acceptance rates of CP recommendations in this study are consistent with the literature [7,15,16]. Notably, in this study, the number of DRPs per patient and the proportion of patients with detected DRPs were higher than those conducted in internal medicine and chest wards. Patients with PE had, on average, one to two more DRPs than those in chest and internal medicine wards. Anticoagulant agents, particularly those used in PE treatment, are the primary contributors to the most frequently observed DRPs in this patient population (Figure 3). The higher number of DRPs in PE patients, despite similar comorbidities observed in the chest and internal medicine wards, is attributed in this study to anticoagulants causing pDDIs and necessitating treatment changes. Variations in study settings, DRP classification systems, and the limited number of DRP studies in PE patients make direct comparisons challenging. However, the inclusion of PE-specific anticoagulants in the treatment regimen underscores the need for vigilant monitoring of patients for DRPs.

Our findings align with prior studies highlighting the prevalence of DRPs in inpatient and outpatient settings. However, compared to studies conducted in internal medicine and chest wards, this study observed a higher number of DRPs per patient, particularly due to the complexity of managing anticoagulants in PE patients. While prior studies predominantly reported DRPs arising from drug selection and dose selection, our findings highlight the additional challenge of managing pDDIs and bleeding risks specific to anticoagulants. These differences emphasize the unique clinical considerations in PE management and the necessity for CP involvement in addressing these challenges.

The DRPs identified in this study and the interventions implemented based on CP recommendations align with findings in the existing literature. The predominant use of LMWHs by patients with PE is noted in this study. However, it is essential to highlight that, apart from specific accompanying indications in PE patients, long-term use of LMWHs may not be warranted [1,2,3]. Recommendations for transitioning to alternative treatment options (VKAs, NOACs) and discontinuing LMWH treatment constitute the majority of interventions in this study. Furthermore, challenges related to the availability of the appropriate dose form of LMWHs (enoxaparin) in Türkiye contribute to difficulties in prescribing LMWHs at the correct therapeutic dose, resulting in DRPs related to underdosing. Additionally, the cost of NOACs in Türkiye and the specific rules set by the Ministry of Health for coverage by health insurance pose challenges. The high acceptance but low implementation rate of CP recommendations in this study can be partially attributed to the nature of the proposed interventions, which primarily involved medication initiation or discontinuation (Figure 2; Figure 3). The implementation of such recommendations was often not feasible solely within the pulmonology outpatient clinic. Given that many patients had multiple comorbidities, executing these recommendations frequently required consultation with other specialties. However, addressing this interdisciplinary coordination within the study design would have introduced significant methodological complexities. Therefore, for the purposes of this study, we assessed the acceptance and implementation rates based only on the decisions made by the pulmonologist, without accounting for potential multidisciplinary consultations. These factors contribute to DRPs, and the implementation rate of CP recommendations for DRPs remains relatively low.

External factors, such as the cost and accessibility of NOACs, play a significant role in influencing patient outcomes and the success of CP interventions. In Türkiye, the high cost of NOACs and restrictive reimbursement policies limit their widespread use, often resulting in the prolonged use of LMWHs. This scenario increases the risk of DRPs, particularly related to dosing errors and pDDIs, as seen in this study. Addressing these systemic barriers is critical to enhancing the effectiveness of CP interventions and ensuring optimal medication management for PE patients.

In the majority of CP studies focused on identifying and resolving DRPs through recommendations, DRPs commonly arise from issues related to drug selection and dose selection. Specifically, DRPs associated with drug selection are often attributed to pDDIs [11,12,13,14]. Notably, in this study, the incidence of DRPs caused by pDDIs appears to be lower than what is typically reported in the literature. Although pDDIs were identified, and warnings related to increased bleeding risk and antiplatelet/anticoagulant effects were prevalent, these warnings did not exhibit significant clinical significance. It is crucial to emphasize that while checking for pDDIs through drug–drug interaction programs is an essential step in mitigating and preventing DRPs stemming from drug selection, the subsequent evaluation of clinical significance and potential risk factors is equally important. Without this evaluation, modifications to drug regimens and other changes may impact treatment unnecessarily.

Studies focusing on clinical pharmacy services explicitly tailored for PE patients and associated DRPs are limited in the existing literature. However, there are related studies demonstrating the impactful role of CP in the management of VTE prophylaxis, which shares some common ground with PE management [17,18,19,20]. One study implemented a standardized VTE prophylaxis protocol developed by CPs in a surgical ward. This protocol included a specialized software application suggesting dose adjustments for obese patients and those with renal impairment. The introduction of this system resulted in a more uniform and rational prescription of pharmacological and mechanical prophylaxis, leading to financial savings. The study emphasized the positive impact of CPs on improving the quality of hospital care [18]. In a CP-led trial conducted by Lee et al. in surgical and medical wards, an increase in the rates of appropriate VTE prophylaxis among patients was reported, along with achieved cost savings [17]. Another study investigated the influence of pharmacist-provided anticoagulation management on mortality, length of stay, and hemorrhage complications in hospitalized patients. Hospitals without pharmacist-provided heparin therapy management had higher mortality rates, increased length of stay, and more bleeding complications compared to those with pharmacist involvement [21]. Haga et al. conducted a study on implementing a clinical pharmacy service, including VTE prophylaxis and promoting appropriate medicine use in the hospital setting. The CP’s interventions ranged from including prophylactic agents, adjusting doses and posology, altering the route of administration, and switching prophylactic drugs, to interrupting prophylaxis to prevent therapeutic duplication [22]. Collectively, these studies highlight the valuable contributions of CPs in improving patient outcomes and optimizing medication management in conditions related to PE and VTE.

In this study, patients in the IG, where the CP provided recommendations for DRPs, did not experience bleeding at the 90-day follow-up. At the 180-day follow-up, bleeding, readmission to the hospital at 90- and 180-day follow-up, difficulty walking, shortness of breath, and pain at 180-day assessment were observed at a lower rate in the IG than in the CG, although the results were not statistically significant (Table 2). A similar before–after comparative study was conducted where a pharmacist was included on the PE intervention team. Major bleeding events decreased when the pharmacist was included on the team, from 14.6% before pharmacist participation to 4.6% after (*p* = 0.0013). Groth et al. emphasized the active role of pharmacists on the PE intervention team, leading to shorter anticoagulation duration, increased use of LMWHs, and fewer major bleeding events [23]. This study, involving a CP in the optimization of PE patients’ treatment, demonstrates a reduction in treatment-related complications and a contribution to the quality of life for patients. The inclusion of CPs on the team during PE patients’ treatment and follow-up processes is suggested based on these findings. This collaborative approach could enhance patient outcomes and safety.

In this study, patients in the IG had a lower median number of comorbidities than those in the CG (*p* = 0.028). The median PESI score was also lower in the IG (*p* > 0.05). In PE patients, the 90-day bleeding rates were significantly associated with decreases in the PESI score and the number of comorbidities. Multiple comorbidities are known to increase the incidence of PE [24]. Considering that comorbidities are among the factors that increase the PESI score, it is a natural conclusion that patients have a lower PE risk with decreases in comorbidities and PESI score. Therefore, patients with multiple comorbidities and high PESI scores are considered at a higher risk for PE. On the other hand, patients with few comorbidities and low PESI scores should be carefully monitored for bleeding complications related to PE treatment. This risk stratification based on comorbidities and PESI scores can help tailor treatment approaches and enhance patient safety.

### Strengths and Limitations

This study is the first randomized controlled trial specifically designed to investigate DRPs in patients with PE. Unlike previous research, it comprehensively evaluates not only DRPs related to anticoagulants but also those associated with all medications used by PE patients. This unique and pioneering approach fills a critical gap in the existing literature, emphasizing the value of CP interventions in identifying, managing, and preventing DRPs.

Despite these strengths, several limitations should be noted. The single-center design and relatively small sample size may limit the generalizability of the findings, as the results reflect the specific conditions and practices of one healthcare facility. Additionally, variations in healthcare systems, including differences in payment conditions for treatment options across countries, may influence the applicability of the results. The inclusion of patients willing to participate in a controlled trial may also introduce selection bias, potentially affecting the representativeness of the findings.

Another limitation lies in the predominance of LMWH use among patients during the study period. While this reflects local treatment practices at the time, it does not align with current clinical trends of NOACs being more commonly prescribed. This heterogeneity in anticoagulant use may limit the reproducibility of the findings and underrepresent DRPs related to NOACs. Furthermore, the cost and limited reimbursement of NOACs in Türkiye during the study period likely contributed to their lower usage, restricting the study’s ability to assess DRPs associated with these medications. Future studies with larger sample sizes and a broader representation of anticoagulant types are needed to address these limitations and provide a more comprehensive understanding of DRPs in PE patients.

Finally, the relatively small sample size may have reduced the statistical power to detect significant differences, particularly those associated with NOAC-related DRPs. While the sample size was sufficient to evaluate primary outcomes, larger, multicenter trials are essential to validate these findings and explore variations in DRPs across anticoagulant regimens. Such studies could also enhance the understanding of CP interventions and their role in improving interdisciplinary collaboration and optimizing DRP management in PE patients.

## 5. Conclusions

Patients with PE experience frequent and complex DRPs, particularly related to drug and dose selection in anticoagulation therapy. Despite similar comorbidities and treatment approaches, DRPs appear to be more prevalent in this population compared to those in internal medicine and chest wards. While CP recommendations were widely accepted by the healthcare team, their implementation remained suboptimal. Notably, in the IG with CP involvement, no bleeding was observed at the 90-day follow-up, whereas bleeding incidents did occur in the CG. These findings suggest that the participation of CPs on the PE treatment team positively influences outcomes such as hospitalization rates and quality of life, even in cases where statistical significance is not reached.

Integrating CPs into interdisciplinary clinical teams caring for PE patients can enhance medication safety, optimize anticoagulation therapy, and reduce DRPs. CPs play a crucial role in monitoring for pDDIs, enhancing adherence to evidence-based treatment guidelines, and minimizing treatment-related complications. Healthcare institutions should consider formalizing CP roles within PE management protocols to enhance patient outcomes.

Further multicenter studies with larger sample sizes are needed to evaluate the comprehensive impact of CP interventions on PE management. Additionally, research should focus on evaluating long-term patient outcomes and developing strategies to increase the implementation of CP recommendations. Investigating how healthcare system variations, particularly differences in anticoagulant accessibility and reimbursement policies, influence CP effectiveness could also provide valuable insights for optimizing medication management in PE patients.

## Figures and Tables

**Table 1:** Baseline demographic and clinical characteristics of patients with pulmonary embolism.

Variable	Total (n = 50)	Control Group (n = 24)	Intervention Group(n = 26)	*p*
**Age (years), median (IQR)**	68.5 (52–76.25)	69 (59.25–79)	66.5 (49.75–74.50)	0.252
**Sex, n (%)**				
Male	20 (40)	8 (33.3)	12 (46.2)	0.399
Female	30 (60)	16 (66.7)	14 (53.8)	
**BMI (kg/m^2^), median (IQR)**	27.6 (23.3–34.5)	27.25 (23.04–34.05)	28.45 (24.35–36.30)	0.627
**Comorbidities (ICD-10 code), n (%)**				
Hypertension	21 (42)	13 (54.1)	8 (30.7)	NA
Diabetes mellitus	13 (26)	8 (33.3)	5 (19.2)	
Chronic obstructive pulmonary disease	9 (18)	5 (20.8)	4 (15.3)	
Coronary artery disease	8 (16)	5 (20.8)	3 (11.5)	
Atrial fibrillation	6 (12)	4 (16.6)	2 (7.6)	
Asthma	6 (12)	3 (12.5)	3 (11.5)	
Heart failure	4 (8)	1 (4.1)	3 (11.5)	
Lung cancer	4 (8)	1 (4.1)	3 (11.5)	
Chronic kidney disease	3 (6)	0 (0)	3 (11.5)	
Parkinson’s disease	3 (6)	1 (4.1)	2 (7.69)	
**Number of comorbidities, median (IQR)**	3 (1–3.25)	3 (2–4)	2 (1–3)	0.028
**Charlson Comorbidity Index score, median (IQR)**	4 (2–6)	4 (2.25–6)	4 (2–5.25)	0.867
**Scores**				
**PESI, mean ± SD**	110.6 ± 34.4	111.9 ± 34.5	109.3 ± 34.9	0.792
**Wells score (DVT), median (IQR)**	0 (0–1)	1 (0–1)	0 (0–1.25)	0.204
**Wells score (PE), median (IQR)**	6 (3–15)	3 (3–15)	10.5 (3–25)	0.344
**Geneva score (PE), median (IQR)**	6 (4–8)	6 (4–7)	6 (3.75–8)	0.762
**DASH score, median (IQR)**	2 (2–3)	2 (2–2)	3 (2–3)	0.016
**Geneva score (DVT), n (%)**				
Low risk	8 (16)	5 (20.8)	3 (11.5)	0.456
High risk	42 (84)	19 (70.2)	23 (88.5)	
**sPESI score, n (%)**				
Low risk	6 (12)	3 (12.5)	3 (11.5)	1.00
High risk	44 (88)	21 (87.5)	23 (88.5)	
**Cancer disease, n (%)**				
Yes	8 (16)	4 (16.7)	4 (15.4)	1.00
No	42 (84)	20 (83.3)	22 (84.6)	
**Total length of stay (day), median (IQR)**	10.5 (6–13.25)	12 (5.25–17)	10 (6.75–12.25)	0.711
**Total number of medications, mean ± SD**	7.6 ± 3.7		7.6 ± 4.2	0.981
**eGFR (mL/min/1.73 m^2^/CKD-EPI), median (IQR)**	96.5 (66.5–110.25)	96 (83.25–111.50)	96.5 (53–108)	0.651

BMI: body mass index, DVT: deep vein thrombosis, eGFR: estimated glomerular filtration rate, IQR: interquartile range, NA: Not applicable, PE: pulmonary embolism, SD: standard deviation.

**Table 2:** Analysis of patients’ treatments and outcomes.

Variable	Total (n = 50)	Control Group (n = 24)	Intervention Group (n = 26)	*p*
**Anticoagulation treatment, n (%)**				
VKA	9 (18)	6 (25)	3 (11.5)	0.281
NOAC	14 (28)	4 (16.6)	10 (38.4)	0.119
LMWH	27 (54)	14 (58.3)	13 (50)	0.584
**Bleeding (90th day), n (%)**				
Yes	4 (8)	4 (16.6)	0 (0)	0.046
No	46 (92)	20 (83.3)	26 (100)	
Bleeding * (day 180), n (%)				
Yes	9 (25)	6 (33.3)	3 (16.6)	0.443
No	27 (75)	12 (66.7)	15 (83.3)	
**Readmission to hospital (90th day), n (%)**				
Yes	10 (20)	6 (25)	4 (15.3)	0.90
No	40 (80)	18 (75)	22 (84.6)	
Readmission to hospital * (day 180), n (%)				
Yes	7 (19.4)	5 (27.7)	2 (11.1)	0.402
No	29 (80.6)	13 (72.2)	16 (88.8)	
Difficulty walking * (day 180), n (%)				
Yes	21 (58.3)	12 (66.6)	9 (50)	0.243
No	15 (41.7)	6 (33.3)	9 (50)	
Pain difficulty state * (day 180), n (%)				
Yes	22 (61.1)	14 (77.7)	8 (44.4)	0.120
No	14 (38.9)	4 (22.2)	10 (55.6)	
Shortness of breath * (day 180), n (%)				
Yes	28 (77.7)	15 (83.3)	13 (72.2)	0.443
No	8 (22.9)	3 (16.6)	5 (27.7)	
**Status of treatment (90th day), n (%)**				
Treatment continues	34 (68)	20 (83.3)	14 (23.8)	0.072
Treatment completed	6 (12)	1 (4.1)	5 (19.2)	
Unknown	10 (20)	3 (12.5)	7 (26.9)	
Status of treatment * (day 180), n (%)				
Treatment continues	17 (47.2)	9 (50)	8 (44.4)	1.00
Treatment completed	19 (52,8)	9 (50)	10 (55.5)	
Difficulty administering medication * (day 180), n (%)				
Yes				0.422
No	22 (61.1)	10 (55.5)	12 (66.6)	
	14 (38.9)	8 (44.4)	6 (33.3)	
**Mortality (day 180), n (%)**				
Yes	12 (24)	5 (20.8)	7 (26.9)	0.745
No	38 (76)	19 (79.2)	19 (71.1)	

LMWH: Low molecular weight heparin, NOAC: Non–vitamin K oral anticoagulants, VK A: Vitamin K antagonist. * It was evaluated on 36 patients due to mortality and inability to reach the patients.

**Table 3:** Classification of drug-related problems.

Classification Divisions	Total, n (%)	Control Group, n (%)	Intervention Group, n (%)	*p*
**Problems**				
P1.2 Effect of drug treatment not optimal	39 (23)	17 (20.2)	22 (26.5)	0.458
P1.3 Untreated symptoms or indication	29 (17.4)	17 (20.2)	12 (14.4)	0.404
P2.1 Adverse drug event (possibly) occurring	39 (23)	19 (22.6)	20 (24.0)	0.757
P3.1 Unnecessary drug treatment	54 (32)	27 (32.1)	27 (32.5)	0.407
P3.2 Unclear problem/complaint. Further clarification necessary	8 (4.7)	6 (0.7)	2 (2.4)	0.175
**The Causes (including possible causes for potential problems)**				
**1. Drug selection**	84 (50.2)	41 (48.8)	43 (51.8)	
C1.1 Inappropriate drug according to guidelines/formulary	8 (4.7)	4 (4.7)	4 (4.8)	0.918
C1.2 No indication for drug	38 (22.7)	19 (22.6)	19 (22.8)	0.635
C1.3 Inappropriate combination of drugs or drugs and herbal medications	16 (9.4)	10 (11.9)	6 (7.2)	0.957
C1.4 Inappropriate duplication of therapeutic group or active ingredient	4 (2.3)	1 (1.1)	3 (3.6)	0.543
C1.5 No or incomplete drug treatment in spite of existing indication	18 (10.7)	7 (8.3)	11 (13.2)	0.429
**2. Drug form**	1 (0.5)	1 (1.1)	0 (0)	
C2.1 Inappropriate drug form/formulation (for this patient)	1 (0.5)	1 (1.1)	0 (0)	0.298
**3. Dose selection**	37 (22.1)	17 (20.2)	20 (24.0)	
C3.1 Drug dose too low	22 (13.1)	9 (10.7)	13 (15.6)	0.305
C3.2 Drug dose of a single active ingredient too high	3 (1.5)	0 (0)	3 (3.6)	0.089
C3.3 Dosage regimen not frequent enough	2 (1)	1 (1.1)	1 (1.2)	0.954
C3.4 Dosage regimen too frequent	9 (5.3)	6 (0.7)	3 (3.6)	0.352
C3.5 Dose timing instructions wrong. Unclear or missing	1 (0.5)	1 (1.1)	0 (0)	0.298
**4. Treatment duration**	2 (1)	1 (1.1)	1 (1.2)	
C4.1 Duration of treatment too short	1 (0.5)	1 (1.1)	0 (0)	0.298
C4.2 Duration of treatment too long	1 (0.5)	0 (0)	1 (1.2)	0.337
**5. Dispensing**	**2 (1)**	**0 (0)**	**2 (2.4)**	
C5.1 Prescribed drug not available	1 (0.5)	0 (0)	1 (1.2)	0.337
C5.2 Necessary information not provided or incorrect advice provided	1 (0.5)	0 (0)	1 (1.2)	0.337
**7. Patient related**	27 (16.1)	10 (11.9)	17 (20.4)	
C7.1 Patient intentionally uses/takes less drug than prescribed or does not take the drug at all for whatever reason	2 (1)	2 (2.3)	0 (0)	0.137
C7.4 Patient decides to use unnecessary drug				
C7.7 Inappropriate timing or dosing intervals	8 (4.7)	4 ()	4 (4.8)	0.934
C7.8 Patient unintentionally administers/uses the drug the wrong way	1 (0.5)	0 (0)	1 (1.2)	0.337
	16 (9.4)	4 (4.7)	12 (14.4)	0.127
**8. Patient transfer related**	3 (1.5)	3 (3.5)	0 (0)	
C8.1 Medication reconciliation problem	3 (1.5)	3 (3.5)	0 (0)	0.298
**Total DRP**	**167 (100)**	**84 (100)**	**83 (100)**	0.601

C: cause, DRP: drug-related problem, P: problem.

**Table 4:** Frequency distribution of potential drug–drug interactions according to study groups.

Interaction Pair	Frequency (Control Group)	Frequency (Intervention Group)	Explanation	Interaction Level	Level of Evidence
Apixaban–dexketoprofen	0	1	Increased antiplatelet effect	D	Fair
Leflunomide–rituximab	1	0	Increased immunosuppressive effect	D	Fair
Enoxaparin–fluoxetine	2	0	Increased antiplatelet effect	D	Fair
Dexketoprofen–warfarin	1	0	Increased antiplatelet effect	D	Fair
Dexketoprofen–fluoxetine	1	0	Increased antiplatelet effect	D	Fair
Diclofenac–enoxaparin	1	0	Increased anticoagulant effect	D	Fair
Escitalopram–rasagiline	1	0	Risk of serotonin syndrome	X	Fair
Dexketoprofen–enoxaparin	0	1	Increased anticoagulant effect	D	Fair
Enoxaparin–sertraline	1	1	Increased antiplatelet effect	D	Fair
Enoxaparin–escitalopram	2	0	Increased antiplatelet effect	D	Fair
Pantoprazole–cefuroxime	1	0	Cefuroxime absorption may be decreased.	X	Fair
Calcium carbonate–methylprednisolone	0	1	The bioavailability of methylprednisolone may be reduced.	D	Fair
Ciprofloxacin–theophylline	0	1	Serum concentration of theophylline may increase.	D	Excellent

## Data Availability

The data will be shared upon requested from corresponding author.
